# A Lynch Syndrome Family With Germline *MLH1* c.931A>G Showing Preserved Tumor MMR Immunostaining but MSI‐H Status: A Case Report

**DOI:** 10.1002/ccr3.73486

**Published:** 2026-09-09

**Authors:** Jingjing Cai, Zhuoying Li, Tingting Han, Yinjin Guo, Yaxi Du, Dan Liu, Xin Liu

**Affiliations:** ^1^ Molecular Diagnostic Center The Third Affiliated Hospital of Kunming Medical University, Yunnan Cancer Hospital, Peking University Cancer Hospital Yunnan Kunming China

**Keywords:** colorectal cancer, likely pathogenic variant, Lynch syndrome, microsatellite instability, mismatch repair, *MLH1*

## Abstract

Lynch syndrome screening may be complicated by discordant tumor testing results. We report a family carrying the germline *MLH1* c.931A>G (p.Lys311Glu) variant in which both the proband and his father had colorectal tumors with retained mismatch repair protein expression by immunohistochemistry but microsatellite instability‐high status on tumor sequencing. The proband was a 39‐year‐old man with colorectal cancer and a family history fulfilling Amsterdam criteria. Paired tumor‐blood sequencing identified MSI‐H, high tumor mutational burden, and the germline *MLH1* variant. The father showed a similar molecular profile and achieved substantial lesion regression after immunotherapy. Family studies demonstrated segregation of the variant across three generations. This case highlights that preserved tumor MMR immunostaining does not exclude Lynch syndrome when clinical suspicion is high. Concurrent MSI and MMR immunohistochemistry, interpreted together with tumor sequencing and germline testing, may improve diagnosis, treatment selection, and familial risk assessment.

## Key Clinical Message

Discordant tumor testing may complicate Lynch syndrome detection. We report a family with an *MLH1* c.931A>G (p.Lys311Glu) germline variant in which both the proband and his father showed proficient MMR by immunohistochemistry but MSI‐high (MSI‐H) on tumor sequencing. Concurrent MSI and MMR immunohistochemistry may improve Lynch syndrome recognition and treatment stratification.

## 1 Introduction

Lynch syndrome (LS) is an autosomal dominant hereditary cancer syndrome that accounts for approximately 1%–3% of newly diagnosed colorectal cancers (CRC) [1]. It is caused by pathogenic germline variants in the DNA mismatch repair (MMR) genes *MLH1*, *PMS2*, *MSH2*, and *MSH6*, as well as deletions involving *EPCAM* [2, 3]. The lifetime risk of CRC and other LS‐associated cancers increases substantially in affected individuals and may reach 50%–80% [4]. If these patients are diagnosed after metastasis, the 5‐year survival rate is about 10.6% [5]. Therefore, early identification enables surveillance and improves outcomes.

Screening for LS may use clinical criteria, including the Amsterdam criteria or revised Bethesda criteria (RBC). But these criteria are too stringent, and if the more sensitive revised Bethesda guidelines had been used, about 10% of variant carriers would have been missed, mostly patients with CRC diagnosed between age 50 and 60 years [6]. Current guidelines recommend universal tumor‐based screening for LS in CRC using MMR immunohistochemistry (IHC) and/or microsatellite instability (MSI) testing [2]. MMR IHC is widely used because of its practicality and lower cost. Nonetheless, reported false‐negative rates for IHC are approximately 5%–10% [7], and the discordance between MSI and MMR IHC results has been reported at roughly 1.7%–10% [8]. Consequently, relying on a single assay may miss a subset of LS. A definitive diagnosis of LS is established by germline testing that identifies pathogenic or likely pathogenic variants in MMR genes. However, some variants are defined as variants of uncertain significance (VUS) [9] due to insufficient research evidence. Even with a suggestive family history, patients with VUS may be overlooked.

Here, we report a family meeting Amsterdam criteria in whom both the proband and his father had colorectal tumors with proficient MMR (pMMR) by IHC but microsatellite instability‐high (MSI‐H) on tumor sequencing, and both carried the germline *MLH1* c.931A>G (p.Lys311Glu) variant. This case highlights the diagnostic and clinical value of integrating family history, tumor IHC, MSI analysis, tumor sequencing, and germline testing in suspected LS.

## 2 Case History/Examination

The patient was a 39‐year‐old man who presented to a local hospital in July 2022 with abdominal pain. His past medical history was unremarkable, with no chronic diseases such as hypertension and no prior surgical procedures.

Colonoscopy revealed a cauliflower‐like mass in the descending colon, approximately 65 cm from the anal verge. The pathological results showed high‐grade dysplasia in some glands, with cancer pending diagnosis. One month later, he went to a tumor specialist hospital for further treatment. Colonoscopy showed that new lesions had developed in the transverse colon. The proband subsequently underwent surgery for colorectal cancer.

## 3 Differential Diagnosis, Investigations, and Treatment

Given the patient's young age at onset and the strong family history suggestive of Lynch syndrome, the differential diagnosis included sporadic colorectal cancer and Lynch syndrome‐associated colorectal cancer. MMR immunohistochemistry and paired tumor‐blood next‐generation sequencing were performed in the proband. Because the family history was highly suggestive of Lynch syndrome, the patient's father also underwent molecular testing.

**FIGURE 1:**
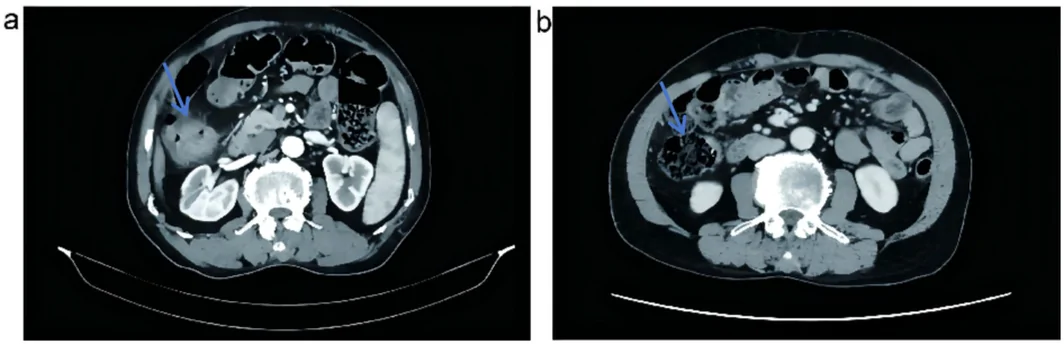
CT scan of the proband's father showed considerable tumor regression. (a) Before treatment, (b) After treatment. Blue arrows indicate the primary lesion(s) in each panel to show tumor size and extent before and after immunotherapy.

In the patient's father, MSI‐H status supported the use of immunotherapy, and the lesion regressed substantially after one year of treatment, indicating clear clinical benefit (Figure 1). In the proband, postoperative infection precluded immediate immunotherapy. Thus, the direct treatment consequence of the discordant pMMR/MSI‐H profile differed between the two affected family members. For the proband, the MSI‐H result did not lead to immediate immunotherapy but remained critical for recognizing LS, refining future therapeutic options if needed, and triggering cascade testing in the family.

## 4 Conclusion and Results

**FIGURE 2:**
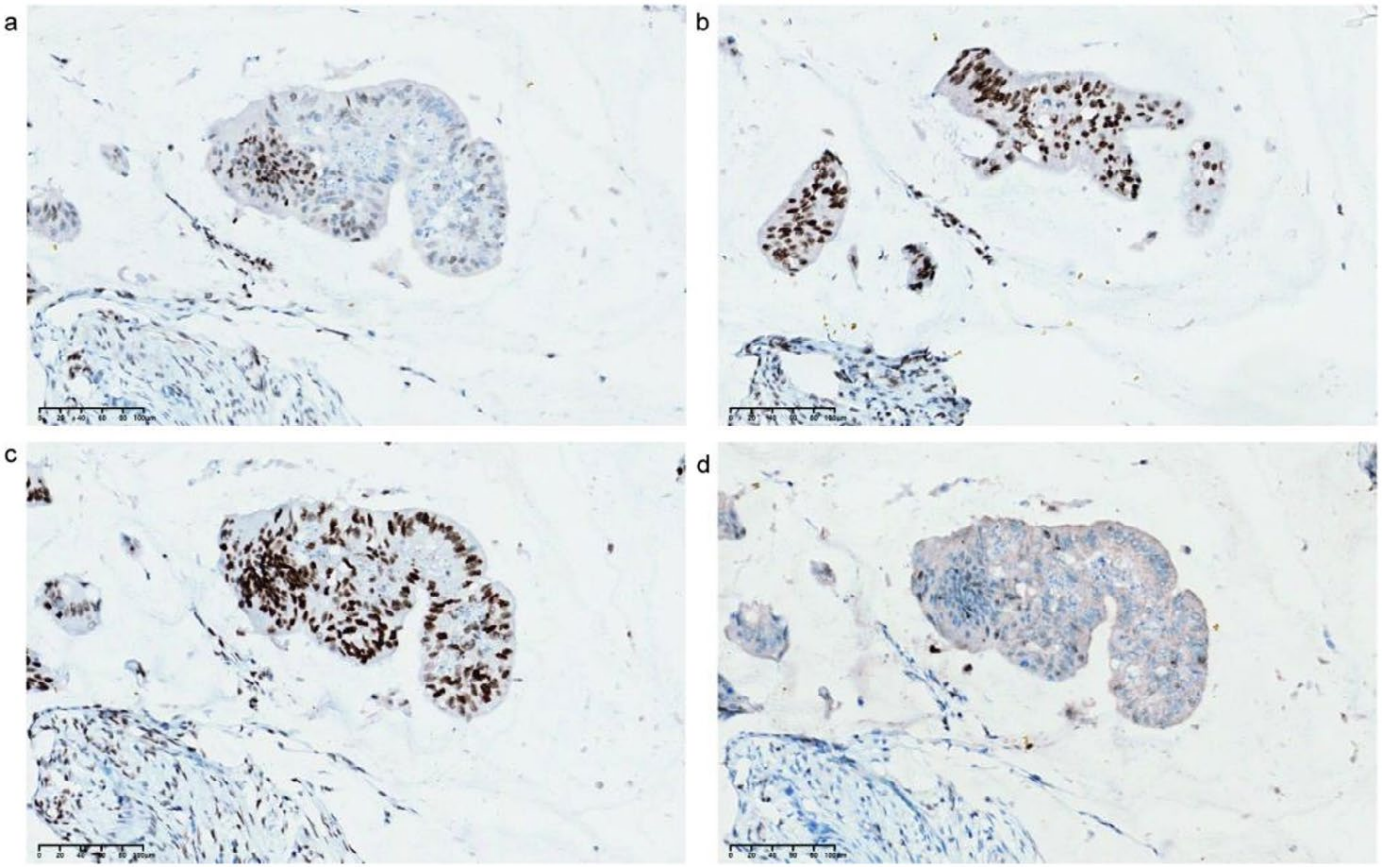
MMR IHC of the proband's tumor (a) MLH1, (b) MSH2, (c) MSH6, and (d) PMS2. Tumor cells showed retained nuclear expression of MLH1, MSH2, MSH6, and PMS2, consistent with a proficient mismatch repair (pMMR) phenotype.

MMR immunohistochemistry of the proband's tumor showed retained nuclear expression of all four MMR proteins (MLH1, PMS2, MSH2, and MSH6) in both the biopsy and postoperative specimens, consistent with a pMMR phenotype by IHC (Figure 2). Subsequent tumor NGS, however, demonstrated MSI‐H status, establishing a discordant pMMR/MSI‐H profile.

Given the proband's early‐onset CRC and family history suggestive of LS despite pMMR by IHC, paired next‐generation sequencing of tumor tissue and peripheral blood was performed. This analysis identified a heterozygous *MLH1* c.931A>G (p.Lys311Glu) variant in peripheral blood, supporting a germline origin. Tumor profiling additionally demonstrated MSI‐H and high tumor mutational burden (TMB‐H). No *BRAF V600E* variant was detected. *MLH1* promoter methylation analysis was not performed. The patient's father also underwent paired tumor–blood sequencing, which similarly showed MSI‐H and TMB‐H. No *BRAF V600E* variant was detected in his tested sample either.

**FIGURE 3:**
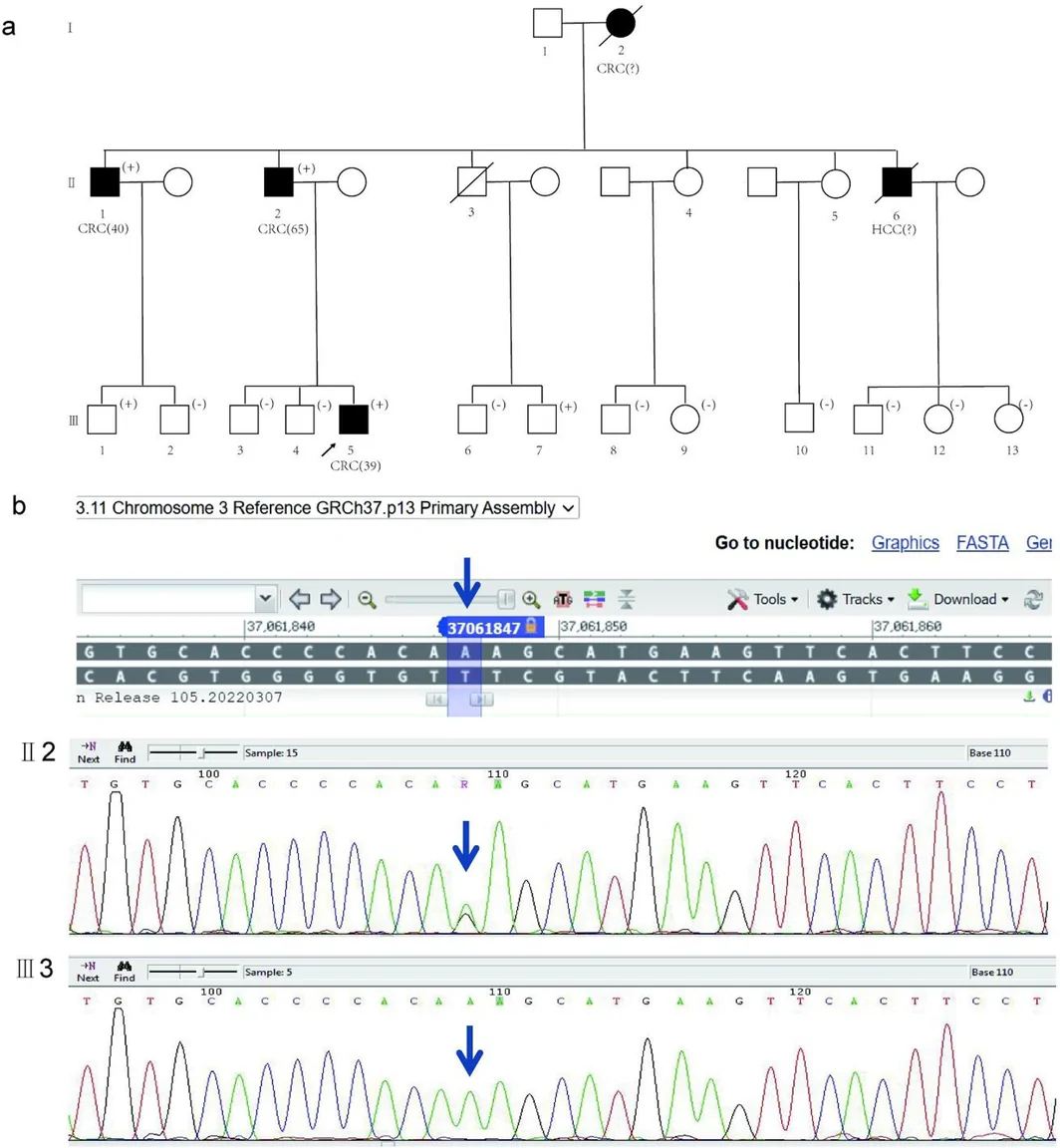
Pedigree and Sanger sequencing of the family. (a) Partial pedigree of the family. Open circles indicate unaffected females, open squares indicate unaffected males, and filled squares indicate individuals with colorectal cancer. The number in parentheses indicates the age at diagnosis. The proband is indicated by an arrow. (+), carrier of the MLH1 variant; (−), non‐carrier. (b) Reference sequence view and Sanger sequencing electropherograms showing the MLH1 c.931A>G variant in family members. The variant position is indicated by blue arrows. Individuals II‐1, II‐2, III‐1, III‐5, and III‐7 carried the variant, whereas the tested non‐carriers did not. HCC, hepatocellular carcinoma.

Pedigree analysis showed cancer clustering across three generations. Sanger sequencing identified the same *MLH1* c.931A>G variant in Individuals II‐1, II‐2, III‐1, III‐5, and III‐7. In addition to the proband (III‐5) and his father (II‐2), Individual II‐1 had been diagnosed with colon cancer at 40 years of age (Figure 3). The remaining carriers were asymptomatic at the time of evaluation, although not all had undergone colonoscopic surveillance. These findings support familial segregation of the variant with LS‐associated cancer.

## 5 Discussion

Lynch syndrome is caused by a variant in the MMR genes, which results in the loss of MMR protein function and deficient mismatch repair activity. Lynch syndrome was previously screened using the Amsterdam and Bethesda criteria; currently, MSI status and MMR protein assessment are widely used. In our case, three generations of the family have Lynch‐associated tumors, with two individuals diagnosed before age 50, meeting Amsterdam criteria. Both the proband and his father were MSI‐H, and the father derived clinical benefit from immunotherapy. Taken together, these findings support the classification of this family as having Lynch syndrome.

The confirmed diagnosis of LS is based on germline testing for pathogenic/likely pathogenic variants. Approximately 50% of Lynch variants occur in *MLH1*, 40% in *MSH2*, and 7% in *MSH6*. The majority of MMR gene variants are small insertions, deletions, or nucleotide substitutions that result in an early stop codon, or nucleotide changes that alter mRNA splicing at the consensus splice site. These variants encode a shorter protein and have been designated as harmful variants, which could help LS diagnosis. By contrast, the clinical significance of many missense variants remains uncertain; such changes are often designated as VUS and may comprise up to 30% of detected alterations in MMR genes [10]. The clinical significance of these genes can only be proven through the accumulation of evidence such as functional experiments and family co‐segregation.

In this study, we identified a germline missense variant, *MLH1* c.931A>G, causing the amino acid at position 311 of the gene encoding protein to mutate from lysine to glutamate. The frequency of this variant in the AMR population of the gnomADe database is 0.00002891 (1/34590), with no records in 1000G, ExAC, or gnomADg databases. Based on the integrated prediction tool REVEL, the variant is predicted to be disease‐causing. This variant has been submitted to ClinVar (ID: 230595) as likely pathogenic with a three‐star review status based on expert panel assessment, including review by the International Society for Gastrointestinal Hereditary tumors (InSiGHT); however, there are no experimental data demonstrating impact on protein function and no published reports of c.931A>G in individuals with Lynch syndrome. Therefore, we describe *MLH1* c.931A>G (p.Lys311Glu) as a likely pathogenic germline variant rather than as a simple variant of uncertain significance.

*MLH1* is a 756‐amino‐acid, 84‐kDa protein that is divided into two parts: the C‐terminal domain (CTD), where *MLH1* paralog dimerization occurs, and the N‐terminal domain (NTD), which has the ATPase activity [11]. The *MLH1* and *PMS2* paralogs produce a MutLα heterodimeric complex, with *MLH1* predominantly being responsible for this interaction [12]. The MutSα mismatch‐recognition complex (*MSH2* and *MSH6* heterodimer) identifies a lesion and recruits MutLα to promote excision and replicative repair in an ATP‐dependent way [13, 14]. *MLH1*'s capacity to interact with adenine nucleotides is a critical element in MMR, causing substantial conformational changes in the protein and impacting the interactions of MutLα with other proteins [15]. Amino acid changes in *MLH1* that affected ATP binding impaired MMR through the *MLH1* null mutant, as shown by instability at microsatellite sequences [16, 17, 18]. *MLH1* homologs are important components of MMR [19]. The ATPase domain (residues 228–336) of *MLH1* folds to form a small α/β barrel at the hLN40 C‐terminus, known as the transducer domain [20]. The residues 298–320 form the ‘QTK’ loop, which has been proposed to act as an ATP sensor. The QTK loop helps to couple changes in ligand binding and hydrolysis to rigid‐body movements and conformational changes in the transducer domain. Within that, Lys311 should function as the conserved basic, ATP gamma‐phosphate‐sensing residue [14]. When the amino acid at this position shifts from lysine to glutamate, protein function changes. Therefore, all prediction algorithms indicate the harmfulness of this locus, and our case provides evidence of co‐segregation, which supports the harmfulness of this variant.

The NCCN Guidelines recommend universal MMR screening of all CRCs and endometrial cancers to maximize sensitivity for identifying LS. Screening modalities include MMR IHC and/or MSI analysis, and genetics consultation is not required prior to routine tumor testing. IHC directly reflects the in situ expression status of MMR proteins, whereas MSI represents a downstream molecular feature of mismatch repair deficiency. NGS can simultaneously assess multiple molecular markers, including MSI and TMB, while peripheral blood germline sequencing is used to determine whether the tumor is associated with hereditary susceptibility [21, 22]. For CRC, MSI has slightly higher sensitivity than IHC for LS detection (92.9% vs. 88.9%–92.4%, respectively), but it is more often not feasible (e.g., due to limited tumor tissue) compared with IHC (14% vs. 0.3%, respectively) [7]. Large studies of universal LS screening have shown that combined tumor testing improves case ascertainment compared with either modality alone, particularly in unusual or technically challenging cases [23]. Reports of MSI‐H/pMMR colorectal cancers have documented clinically significant subsets of patients who might otherwise be missed by IHC‐only screening [24], including some with germline MMR variants and others with treatment‐relevant hypermutated tumors. In precision oncology practice, broad tumor NGS panels frequently include MSI and TMB assessment, allowing simultaneous evaluation of inherited cancer suspicion, tumor biology, and eligibility for immunotherapy.

This case represents an uncommon but clinically important example of retained MMR protein expression by IHC combined with MSI‐H status. Both the proband and his father showed retained expression of all four MMR proteins on IHC and were initially classified as pMMR; however, tumor sequencing subsequently revealed MSI‐H. Previous studies have described several mechanisms that can underlie discordant pMMR/MSI‐H results. Missense variants (e.g., the MLH1 p.Lys311Glu reported here), or indels in non‐coding regions, may abrogate MMR function while preserving epitopes recognized by diagnostic antibodies; in such cases IHC may be positive despite an MSI‐H tumor [25]. Tumor subclonality and molecular heterogeneity can also produce focal MMR protein loss confined to a subset of cells, which may be missed on routine assessment and give a false impression of preserved expression [26]. Alterations in less commonly assessed MMR genes (e.g., *MSH3*) can likewise drive MSI‐H and produce a pMMR/MSI‐H profile [27]. Finally, specimen processing and fixation methods, antibody clone types and detection sensitivity, as well as subjective interpretation by the reporting pathologist, may further lead to inconsistent test results. Based on the MSI results, the father was able to initiate immunotherapy and achieved marked regression of the lesion. The proband missed the opportunity for early immunotherapy because of postoperative infection, but the MSI‐H result nevertheless corrected the potential false‐negative interpretation caused by reliance on IHC alone. This not only supported the diagnosis of Lynch syndrome and guided long‐term individualized follow‐up, but also facilitated cascade genetic screening among family members. Previous studies have reported discordance rates between IHC and MSI results ranging from 1.7% to 10% [8], which is consistent with the findings in this case and highlights the clinical limitations of using a single testing method. Based on this evidence, simultaneous MMR‐IHC and MSI testing is recommended whenever clinically feasible, to reduce missed diagnoses, optimize antitumor treatment strategies, and improve familial risk management.

In conclusion, we report a Lynch syndrome family carrying the likely pathogenic *MLH1* c.931A>G (p.Lys311Glu) germline variant in which both the proband and his father had retained MMR protein expression by IHC but MSI‐H tumors on sequencing. This case demonstrates that intact MMR immunostaining does not exclude LS or MMR dysfunction when clinical suspicion is high. Concurrent MSI testing and MMR IHC, interpreted together with family history, tumor sequencing, and germline analysis, may reduce missed diagnoses, identify treatment opportunities, and improve familial risk assessment.

## Author Contributions

**Yaxi Du:** investigation, project administration. **Yinjin Guo:** investigation, methodology. **Tingting Han:** investigation, methodology, writing – original draft, visualization. **Dan Liu:** investigation, visualization, validation, data curation. **Zhuoying Li:** investigation, methodology, data curation, resources. **Xin Liu:** conceptualization, writing – review and editing, supervision, resources, project administration, funding acquisition. **Jingjing Cai:** investigation, data curation, formal analysis, writing – original draft, validation.

## Funding

Medical Academic Leader Training Program of Yunnan Provincial Health Commission (Grant D‐2019028), National Natural Science Foundation of China (Grant 81860485), the Applied Basic Research Foundation of Yunnan Province Science and Technology Department (Grant 202301AT070246), Yunnan Provincial Academician Zhan Qimin Workstation (Grant CZGZZ202405AF140011).

## Consent

Written informed consent was obtained from the proband and all participating family members for genetic testing and for publication of this case report and accompanying images.

## Conflicts of Interest

The authors declare no conflicts of interest.

## Data Availability

Data sharing is not applicable to this article as no datasets were generated or analyzed beyond the clinical data reported in the manuscript. De‐identified individual‐level data are available from the corresponding author on reasonable request and with appropriate approvals.
